# The Potential Role of MUC16 (CA125) Biomarker in Lung Cancer: A Magic Biomarker but with Adversity

**DOI:** 10.3390/diagnostics12122985

**Published:** 2022-11-29

**Authors:** Hebatallah M. Saad, Ghada F. Tourky, Hayder M. Al-kuraishy, Ali I. Al-Gareeb, Ahmed M. Khattab, Sohaila A. Elmasry, Abdulrahman A. Alsayegh, Zaki H. Hakami, Ahmad Alsulimani, Jean-Marc Sabatier, Marwa W. Eid, Hazem M. Shaheen, Ali A. Mohammed, Gaber El-Saber Batiha, Michel De Waard

**Affiliations:** 1 Department of Pathology, Faculty of Veterinary Medicine, Matrouh University, Marsa Matruh 51744, Matrouh, Egypt; 2 Faculty of Veterinary Medicine, Damanhour University, Damanhour 22511, AlBeheira, Egypt; 3 Department of Clinical Pharmacology, Internal Medicine, College of Medicine, Al-Mustansiriyiah University, Baghdad P.O. Box 14132, Iraq; 4 Pharmacy College, Al-Azhar University, Cairo 11884, Cairo, Egypt; 5 Faculty of Science, Damanhour University, Damanhour 22511, AlBeheira, Egypt; 6 Clinical Nutrition Department, Applied Medical Sciences College, Jazan University, Jazan 82817, Saudi Arabia; 7 Medical Laboratory Technology Department, College of Applied Medical Sciences, Jazan University, MS, CT (ASCP), PhD, Jazan 45142, Saudi Arabia; 8 Aix-Marseille Université, Institut de Neurophysiopathologie (INP), CNRS UMR 7051, Faculté des Sciences Médicales et Paramédicales, 27 Bd Jean Moulin, 13005 Marseille, France; 9 Department of Pharmacology and Therapeutics, Faculty of Veterinary Medicine, Damanhour University, Damanhour 22511, AlBeheira, Egypt; 10 Consultant Respiratory & General Physician, The Chest Clinic, Barts Health NHS Trust Whipps Cross University Hospital, London E11 1NR, UK; 11 Smartox Biotechnology, 6 rue des Platanes, 38120 Saint-Egrève, France; 12 L’institut du Thorax, INSERM, CNRS, UNIV NANTES, 44007 Nantes, France; 13 Université de Nice Sophia-Antipolis, LabEx «Ion Channels, Science & Therapeutics», 06560 Valbonne, France

**Keywords:** MUC16 (CA125), lung cancer, chemoresistance

## Abstract

Lung cancer is the second most commonly diagnosed cancer in the world. In terms of the diagnosis of lung cancer, combination carcinoembryonic antigen (CEA) and cancer antigen 125 (CA125) detection had higher sensitivity, specificity, and diagnostic odds ratios than CEA detection alone. Most individuals with elevated serum CA125 levels had lung cancer that was either in stage 3 or stage 4. Serum CA125 levels were similarly elevated in lung cancer patients who also had pleural effusions or ascites. Furthermore, there is strong evidence that human lung cancer produces CA125 in vitro, which suggests that other clinical illnesses outside of ovarian cancer could also be responsible for the rise of CA125. MUC16 (CA125) is a natural killer cell inhibitor. As a screening test for lung and ovarian cancer diagnosis and prognosis in the early stages, CA125 has been widely used as a marker in three different clinical settings. MUC16 mRNA levels in lung cancer are increased regardless of gender. As well, increased expression of mutated MUC16 enhances lung cancer cells proliferation and growth. Additionally, the CA125 serum level is thought to be a key indicator for lung cancer metastasis to the liver. Further, CA125 could be a useful biomarker in other cancer types diagnoses like ovarian, breast, and pancreatic cancers. One of the important limitations of CA125 as a first step in such a screening technique is that up to 20% of ovarian tumors lack antigen expression. Each of the 10 possible serum markers was expressed in 29–100% of ovarian tumors with minimal or no CA125 expression. Therefore, there is a controversy regarding CA125 in the diagnosis and prognosis of lung cancer and other cancer types. In this state, preclinical and clinical studies are warranted to elucidate the clinical benefit of CA125 in the diagnosis and prognosis of lung cancer.

## 1. Introduction

Lung cancer is the second most commonly diagnosed cancer in the world [1]. More than two million lung cancer patients were diagnosed in recent years, making up 12% of the total number of cancer cases worldwide [1,2]. With around 1.37 million diagnoses in Europe in 2018, lung cancer is the most prevalent cancer diagnosis in men [3]. Around 725,000 new cases of lung cancer were diagnosed in women in 2018, which is typically a lower incidence rate than in men [3]. Women’s geographic incidence rates differ from men’s, which is attributed to historical variances in cigarette smoking [3,4]. The greatest smoking rates among women in North America and Europe may raise the risk of lung cancer [5]. Lung cancer appears to be the second most frequent cancer in males, following prostate cancer, and in women, following breast cancer [5]. The incidence rate among men is 71.3/100,000 and for women is 52.3/100,000. Due to chronological differences between both sexes in smoking initiation and cessation, the incidence rate for women did not begin to decline until the middle of 2000, even though it has been declining for males since the middle of 1980 [3,5]. Lung cancer incidence has decreased considerably during the past ten years, with rates falling by roughly 3% annually for males and 1.5% annually for women from 2011 to 2015, corresponding with a reduction in smoking habits [6].

Due to poor survival and the high mortalities of lung cancer, the worldwide geographical prototype in lung cancer-related deaths intimately follows the incidence. Lung cancer is the second-leading cause of cancer-related death in women and the first-leading cause in men worldwide [7]. Patients with lung cancer have experienced relatively modest increases in 5-year survival despite significant advancements in survival for the majority of other cancer types in several nations in recent years [4,7]. The main reason for this lack of improvement is due to most patients are identified with late-stage diseases, which have low survival rates [4]. Small cell lung cancer (SCLC) and non-small cell lung cancer (NSCLC) both have a 5-year relative survival rate of 19%, with NSCLC having a better 5-year survival rate (23%) than SCLC (6%) [6].

Notably, pulmonary cancer is divided histologically into NSCLC and SCLC. NSCLC represents 80–85% of lung cancers, of which about 40% is adenocarcinoma, 25–30% is squamous cell carcinoma, and 10–15% is large cell carcinomas [8]. However, bronchioloalveolar carcinoma is a separate histological classification that represents a subdivision of adenocarcinomas and is substituted by pulmonary adenocarcinoma in situ, minimally invasive adenocarcinoma, and invasive adenocarcinoma [9]. Furthermore, the remaining histologic subdivisions are adenosquamous carcinoma, pleomorphic sarcomatoid carcinoma, large-cell neuroendocrine carcinoma, and carcinoid tumor. The most popular histologic subdivision of cancer identified in females is adenocarcinoma [9]. Furthermore, since the 1970s, men have experienced exponential growth in pulmonary adenocarcinoma incidence; this increase has caused it to exceed squamous cell carcinoma in incidence rate [10]. 

Notoriously, since the early 1980s, the incidence rate of squamous cell carcinomas has decreased [11]. This sequential change in histologic diagnoses is substantially attributed to the frequent use of filtered cigarettes and rising levels of tobacco-specific nitrosamines in tobacco [11]. Smoking unfiltered tobacco increases the amount of combusted tobacco smoke-exposed to the trachea and bronchi, resulting in greater rates of squamous cell carcinoma diagnoses, mostly in men [12]. Since the advent of filtered cigarettes, combusted tobacco smoke has disseminated further into the respiratory tree due to deeper inhalation, leading to adenocarcinomas with a greater perivascular distribution [13]. The introduction of light-filtered cigarettes and altering tobacco blends, which reduced nicotine but elevated nitrates and N-nitrosamines, had the counterintuitive consequence of raising rather than lowering the risk of lung cancer because it encouraged longer, more frequent, and deeper inhalations of combusted tobacco smoke [12,13]. 

Later on, in the mid-2000s, electronic cigarettes (e-cigarettes) were used more by both men and women. This enables nicotine delivery to the pulmonary epithelium by an electronic device [14]. Of interest, studies have demonstrated that the two main solvents used in e-cigarettes, vegetable glycerin and propylene glycol, emit vapors that are hazardous and carcinogenic due to the presence of carbonyl chemicals such as formaldehyde, acetaldehyde, acetone, and acrolein [14,15]. Moreover, the using of e-cigarettes is linked to more oxidative stress, which is responsible for the negative consequences of e-cigarettes. Further, e-cigarettes-induced oxidative stress leads to inflammation, cytotoxicity, increased endothelial cell permeability, and the development of lung cancer [14,15]. In addition, e-cigarettes lead to harmful effects on the function of the lung. It has been demonstrated that e-cigarette vapor has harmful compounds that have negative impacts on the health of humans [16]. In contrast, results indicated that e-cigarettes can be a less harmful substitute to traditional cigarettes, although there are no data on the long-term cancer risk associated with low-level exposure to the detected carcinogens from e-cigarettes. As well, the effect of e-cigarettes on the risk of lung cancer must be clarified [17]. In terms of prevention, more studies are required to detect potential factors to decrease the risk of lung cancer, particularly among former smokers [16,17]. 

Despite significant advances in lung cancer control in terms of etiology, prevention, early detection, diagnosis, and treatment, lung cancer remains a major public health burden globally [17]. In this state, many efforts are required to detect causal risk factors for pulmonary tumors among never-smokers and to detect never-smokers at the highest risk for pulmonary tumors who may benefit from a pulmonary tumor screening program [18,19].

Therefore, precision-based risk assessment and screening may be investigated to detect patients who could benefit the most from participating in a pulmonary cancer screening program. Advances in screening technology and biomarkers in the screening setting may reduce false positives and over-diagnosis while also improving nodule management [20]. There is a significant clinical need for biomarkers that are extremely predictive of negative responses to targeted therapies and immunotherapy since certain patient subgroups may not respond to these specific treatments [20].

In terms of the diagnosis of lung cancer, combination carcinoembryonic antigen (CEA) and cancer antigen 125 (CA125) detection had higher sensitivity, specificity, and diagnostic odds ratios than CEA detection alone [21]. The combined detection area under the curve (AUC) was 0.90, while the independently detected AUC was 0.73. Thus, the combined usage of CEA and CA125 is more effective in diagnosing pulmonary tumors than CEA detection alone [21]. The importance of the combined usage of CEA and CA125 in the detection of pulmonary tumors has been established.

Most individuals with elevated serum CA125 levels had lung cancer that was either in stage 3 or stage 4 [22]. Serum CA125 concentrations were similarly increased in lung tumor patients who also had pleural effusions or ascites. Patients with elevated serum CA125 levels had significantly shorter survival times than those with normal levels [22]. Increased concentrations of serum CA125 were found to be a poor prognostic factor in individuals with advanced disease. The prognosis of pleural effusion was not related to survival time. Thus, CA125 is a good predictor of disease progression, and serum levels correlate with survival time in individuals with pulmonary cancer [22]. Furthermore, there is substantial evidence for the production of CA125 in vitro by human lung cancer, implying that the increase in CA125 serum levels may be caused by clinical conditions other than ovarian cancer [23].

Therefore, the present review aimed to find the potential role of CA125 in the diagnosis and follow-up of patients with lung cancer.

## 2. MUC16 (CA 125) as a Lung Cancer Biomarker

CA125 is a carbohydrate epitope that was discovered with a high molecular weight of 2 million Da. Mucin 16 (MUC16) was discovered to be a mucinous glycoprotein with a molecular weight ranging from 3–5 million Da after molecular cloning. Similar to other membrane panning mucins, the MUC16 C-terminal domain, which is made up of 284 amino acids, is the molecule’s smallest component [24]. Although it has been proposed that this C-terminal region can be phosphorylated under certain circumstances, there is no clear evidence. There are 12,068 amino acids in the N-terminal region. There are no noteworthy structural features in this domain other than possible locations for both N-linked and O-linked glycosylation. There are up to 60 tandem repetitions in the tandem repeat domain [25]. There are 156 amino acids in each repeat. Each repeat has a different primary amino acid sequence, although they are all homologous. At positions 59 and 79 of each repeat, two conserved cysteines are thought to have structural relevance [26]. These cysteines can generate intramolecular and intermolecular disulfide connections. While intramolecular disulfide bonds may form loops within each MUC16 molecule, intermolecular disulfide linkages may be involved in the formation of the extracellular matrix [27].

MUC16 (CA125) is a natural killer (NK) cell inhibitor [24,28]. CA125 is widely used as a marker in three separate clinical cases, in which it is considered as a screening test for the early diagnosis of ovarian cancer, to distinguish benign from malignant disease in pre-and postmenopausal women with pelvic masses, and finally to track the therapeutic response in ovarian cancer patients [28]. In pre-menopausal women with ovarian tumors or pelvic masses, CA125 as a biomarker is confounded by the known elevation during menstruation. The CA125 level is transiently increased during menstruation which gives false-positive results [29]. As well, the CA125 level is increased significantly in the non-menstrual part of the cycle in women with advanced endometriosis and adenomyosis [29]. A cohort study found that the menstrual CA125 level was higher than the non-menstrual CA125 level [30]. Therefore, measurement of the CA125 level should be avoided during the menstrual phase to avoid misleading results. These findings suggest that evaluation of the CA125 level is better performed in menopause to exclude the effect of menstruation. Besides, additional tests in combination with the measurement of the CA125 level were performed, like HE4 for the evaluation of ovarian tumors or pelvic masses, and have proposed a risk of malignancy (ROMA) index [31]. Dochez et al. [31] observed that combined measures of HE4 and CA125 were more efficient with high specificity. These combined measures can correct the variations in both HE4 and CA125 by different factors like smoking and menstruation, respectively [31].

Regarding the results, only half of the individuals with stage I ovarian cancer could be detected [28,32]. The identification percentage elevated to roughly 85–94 percent for individuals with ovarian cancer at stage II-IV, making CA125 a helpful marker solely for detecting late stages of ovarian cancer, which limited the biomarker’s purpose. This matched the findings of Kim and colleagues [2], who found MUC16 to be highly mutated in many malignant tumors, such as lung cancer. Likewise, Ma and colleagues [33] studied the predictive usefulness of CA125 (MUC16) and other biomarkers in 164 individuals with stage I NSCLC who had surgery (101 men, 63 women). The researchers discovered a 5.7% positive MUC16 (CA125) result in 131 adenocarcinoma cases and a 3.1% positive CA125 result in 43 non-adenocarcinoma cases. Although MUC16 (CA125) levels were higher, the researchers showed that additional studies are required to prove CA125’s potential as a biomarker in lung cancer. Unlike previous studies, Kanwal and colleagues [34] studied and measured MUC16 mRNA concentrations in NSCLC and adjacent non-malignant tissues in 84 patients (51 men and 33 women) living in China’s air-polluted regions. MUC16 mRNA concentrations were considerably higher in 48.8% (41/84) of NSCLC tissues as compared to adjacent noncancerous tissues. MUC16 mRNA expression, on the other hand, did not connect with gender (*p* = 0.74), age (*p* = 0.27), or histological type (*p* = 0.53). Despite the small sample size, this study yielded promising results, demonstrating that MUC16 upregulation driven by gene mutation may play a role in the formation and progression of pulmonary cancer. MUC16 has been linked to the development and spread of numerous malignancies, although its role in lung carcinoma is unknown. Larger, more rigorous investigations are needed to examine the reproducibility of the findings from the studies discussed earlier in this regard [35].

A competent clinical laboratory used an electro-chemiluminescence immunoassay technology to detect serum CEA and CA125 concentrations in 86 (48 male and 38 female, median age 60.6 years, range 38–76 years) of the 160 individuals [25]. They had a 60.0 percent (5/86) 5-year survival rate. As a result, data on concentrations were collected from electronic medical records. The serum CEA expression level had a cut-off of 5 ng/mL, while the serum CA125 expression level had a cut-off of 35 U/mL. Forty-two (48.8%) of the 86 patients having preoperative serum samples had an elevated serum CEA expression level (5 ng/mL), whereas 19 (22.1%) had a high serum CA125 expression level (35 U/mL). Further, 117 (73.1 percent) of the 160 patients with IHC staining findings had positive signal transducer and activator of transcription 3 (STAT3) expression, 41 (25.6 %) expressed positive Phospho-signal transducer and activator of transcription 3 (pSTAT3) expression, and 55 (34.4 %) expressed positive interleukin (IL)-17 expression. Patients with increased serum CEA or CA125 levels had considerably lower disease-specific survival, according to the findings. All three outcomes (STAT3, pSTAT3, and IL-17) have significant associations with CA125 (*p* 0.05). More research into more effective prognostic indicators is required. There were two key findings in this study. First, the survival analyses revealed that patients with leukocyte adhesion deficiency (LAD) had a lower chance of survival because of the greatest levels of CEA and CA125 in the serum and pSTAT3 and IL-17 in the tumor tissue. The subgroup survival studies further demonstrated that the levels of co-expression of these biomarkers might be used to stratify the clinical outcomes of individuals with postoperative LAD. Second, the correlation tests revealed a significant positive association between the concentrations of CA125 (MUC16), STAT3, pSTAT3, and IL-17; their synergic activities were linked to the Tumor Node Metastasis (TNM) stage.

These results provided the foundation for the predictive value of CA125, STAT3, pSTAT3, and IL-17 in individuals with postoperative LAD. With the Kaplan–Meier survival analysis, there is a significant association between positive CEA, CA125, pSTAT3, and IL-17 expression and poor survival rate after the surgery, but STAT3 expression had no predictive value for patient prognosis [27,28,35]. Following Cox regression analyses, the roles of CEA, CA125, pSTAT3, and IL-17 as independent prognostic predictors in LAD were established [25]. In NSCLC patients, positive results were only seen in CEA, CA125, neuron-specific enolase (NSE), and cytokeratin fraction 21-1 (CYFRA21-1), with positive rates of 22.50 percent, 5.88 percent, 5.88 percent, and 37.93 percent, respectively [26].

Among the 40 cancer and non-cancer disorders studied, the median CA125 serum levels in individuals with pulmonary fibrosis and cirrhosis were 52.04 and 52.34 U/mL, respectively, with the mean value elevated to 165.32 U/mL in cirrhosis and 79.01 U/mL in pulmonary fibrosis. CA125 mRNA is present in the lung, salivary gland, testis, fallopian tube, cervix, uterus, placenta, and skin tissues, according to the Human Protein Atlas website’s database, whereas CA125 protein expression is detected in reproductive glandular cells and respiratory epithelium [27]. According to theory, increased CA125 production and a decreased metabolic clearance rate in blood circulation could be the causes of increased CA125 concentrations in blood circulation. Individuals with pulmonary cancer and ovarian cancer had the greatest 2Log10 *p* values, which was surprising [27]. Patients with idiopathic pulmonary fibrosis (IPF) have significantly higher levels of serum CA125, suggesting that CA125 could be a biomarker for epithelial damage, according to cohort research [36]. Similarly, CA125 levels in the blood are increased in cystic fibrosis patients [27]. In malignant illnesses, hypoxia enhances cancer to grow faster and produce more CA125 [27,37]. More CA125 may be secreted into the bloodstream due to severe cellular injury [38]. Furthermore, malignant disorders frequently cause neovascularization, vascular invasion, and destruction, all of which might result in increased CA125 release into the bloodstream [35]. This may be due to the greatest 2Log10 *p* values found in lung and ovarian malignancies. It may also explain why blood CA125 levels are difficult to utilize for early ovarian cancer detection, because increased CA125 release may not be meaningful at this stage of tumor development [27].

CA125, an oxidoreductase 1–like (ERO1L) downstream molecule, may be a useful biomarker for assessing the efficacy of immunotherapy, which warrants additional investigation. ERO1L has a significant function in the development of lung tumors [39]. First, ERO1L modulates MUC16 expression via cytokine production and stimulates the production of CA125, presenting recent therapeutic possibilities for MUC16 and the usage of numerous tumor indicators in diagnosis. Second, ERO1L is responsible for white blood cell recruitment and modulation of the expression of major histocompatibility complex (MHC) molecule12, allowing CA125 to be used in areas other than lung cancer diagnosis and treatment, such as immunotherapy [40].

Albeit MUC16 expression was upregulated in 51% of smokers [41], the expression of MUC16 mRNA did not show any significant difference between smokers and nonsmokers [41]. In a study of 14 cell lines to see if MUC16 expression was present in cultured lung cancer cells, three cell lines of lung cancer (A549, 801-D, and NCI-H446) had greater MUC16 mRNA concentration than immortal human bronchial epithelial cell lines. For analyzing mutation distribution in the MUC16 gene, 22 tissue samples (10 pairs of NSCLC and their neighboring nonmalignant tissues, in addition to two carcinogenic tissues) and 10 cell lines of lung cancer were chosen for captured target gene sequencing. Total single-nucleotide polymorphism (SNP) and data of insertion and deletion (InDel) from each individual were collected and compared between the two groups [28,34,42]. After all shared SNPs and InDels were removed, the MUC16 up-regulated group and the MUC16 unchanged/down-regulated group displayed distinct patterns. For further investigation, some specific sites and areas with a considerably imbalanced mutation distribution across the two groups were chosen [41]. In prior research, whole-genome sequencing found that several genes had much greater mutation rates and numbers in the cancer of the lung from highly polluted areas than in NSCLCs from control areas. MUC16 mRNA was found to be upregulated in tissue samples and cultured cells treated with the S5 and S5-1 systems. MUC16 upregulation has been linked to tumor cell invasion, aggressiveness, and metastasis in a variety of cancer types. Furthermore, MUC16 can bind to specific cells, including NK cells and monocytes, and cause functional reactions when it is released into circulation. MUC16 upregulation may help in cancer cell protection from cytolysis and the immune response [28,34].

When serum tumor marker concentrations in individuals with several types of lung cancer were compared, researchers discovered that CA125 and CEA concentrations in the pulmonary cancer group were higher than those in the benign group [43]. Patients with adenocarcinoma had greater CA125 and CEA values than those without. CA125 levels in the pulmonary benign and malignant tumor groups were compared. The pulmonary cancer group (*n* = 94) and the benign group (*n* = 26) are represented by the abscissa, while the ordinate reflects the CA125 level (U/mL). The pulmonary cancer group had CA125 values of (34.50 ± 5.23) U/mL, while the benign group had (16.68 ± 7.85) U/mL. *p* < 0.001. CA125 is considered the main biomarker of adenocarcinoma, and the principal reason for increased CA125 levels in lung cancer cases is that the CA125 antigen is induced and then constantly released and eventually enters the bloodstream via autonomous absorption, increasing CA125 concentrations [43,44].

These verdicts suggest that CA125 could be a useful marker for pulmonary cancer diagnosis and prognosis.

## 3. Muc16 (CA 125) and Malignant Proliferation

Lung nodules, NSCLC, SCLC, and mesothelioma are the most common types of pulmonary cancer [45]. Rare lung cancers do not always start in the lungs. The size of recommended treatment choices and the rate of metastasis of rare lung tumors vary [46]. Proliferation refers to the rate at which lung cancer cells multiply. The amount of time it takes for a tumor to double in size is known as doubling time [34,47]. MUC16 is a transmembrane glycoprotein that regulates cell adhesion, protein–protein interactions, and immunology by changing its expression and glycosylation pattern [48]. A mutant MUC16 gene is found in 50% of lung malignancies caused by air pollution. In a large investigation of lung cancer, MUC16 gene mutations were found in 53 percent of cases, including 51 percent of adenocarcinoma and 56 percent of squamous cell carcinoma [49]. MUC16 was considered one of three genes with the greatest frequency of mutations across a variety of cancers [50]. The high MUC16 expression caused by gene mutations affected cell proliferation, migration, and invasion in cultured lung cancer cells in this work. MUC16 overexpression generated by mutations in the MUC16 gene greatly improved cellular proliferation, migration, and invasion capacities. As a result, they believe that MUC16 overexpression caused by gene alterations has functional implications for lung cancer cell behaviors [32]. The formation and progression of cancer are aided by cellular proliferation, migration, and invasion [40].

CA125 is a helpful tumor biomarker in ovarian and lung cancer surveillance [51]. It is a tumor marker that is sensitive but not specific. CA125 levels in the blood are elevated in a variety of benign and malignant disorders [31]. They reported two examples of elevated serum CA125 levels after a thoracotomy for removal of an early-stage lung tumor that spontaneously regressed 3 and 8 months after surgery in this research. CA125 levels in the blood may rise following thoracotomy for reasons unrelated to tumor recurrence. As a result, postoperative CA125 readings should be interpreted with caution [32,52].

According to certain research, MUC16 could be a viable therapeutic target for cancer patients [53]. In one research study based on the Atlas of Cancer Genome, MUC16 is considered one of the most frequently mutated genes (TP53, USH2A, TTN, MUC16) in various malignancies such as lung cancer [32]. MUC16 expression was found in NSCLC patients in China’s Yunnan Province who were affected by familial lung cancer (FLC) and indoor air pollution caused by coal use, as well as the study looked into the role of MUC16 in lung cancer cell proliferation, migration, invasion, and chemosensitivity. There are several studies in this category [34,40]. Clinic pathologic features and MUC16 expression were investigated and evaluated [40]. MUC16 gene knockout and overexpression vectors and then looked at how MUC16 affected lung malignant cell characteristics like proliferation, migration, and invasion. FLC was found to have a significant relationship with early-onset (P0.01) and later-stage (P0.01) [53]. Finally, MUC16 plays an important role in lung cancer development, progression, and chemo-resistance [54]. Its link to FLC and indoor air pollution, for example, underscores the complexities of lung cancer causation [53].

SCLC accounts for about a third of all new lung cancer cases or around 180,000 cases each year worldwide [1]. Changes in smoking habits have reduced the frequency of SCLC over the last 30 years, primarily in developed countries such as Canada and the United States. In Eastern Europe and Asia, on the other hand, the incidence of SCLC has grown because of the sustained high prevalence of smoking [1,19,55]. SCLC is considered a malignant tumor of epithelial origin composed of tiny cells with sparse cytoplasm, poor-defined cellular boundaries and the nucleus contains finely granular chromatin with absent or inconspicuous nucleoli, as illustrated by Van Meerbeeck and colleagues [56]. Only small cells are implicated in 90% of instances, while giant cell components are present in the remaining cases [19]. SCLC stages are often classified as modest or extensive. A tumor that is contained within the pulmonary tissue, but has not spread as far as diagnostic assessment can establish, is known as a limited-stage disease [57]. The tumor has progressed to the contralateral lung, distant lymph nodes, and/or other bodily organs. SCLC is distinguished by the following characteristics: it is an aggressive tumor with early metastasis, it has a high spreading and proliferation, and it has a good early response to chemotherapeutics [58].

**Figure 1:**
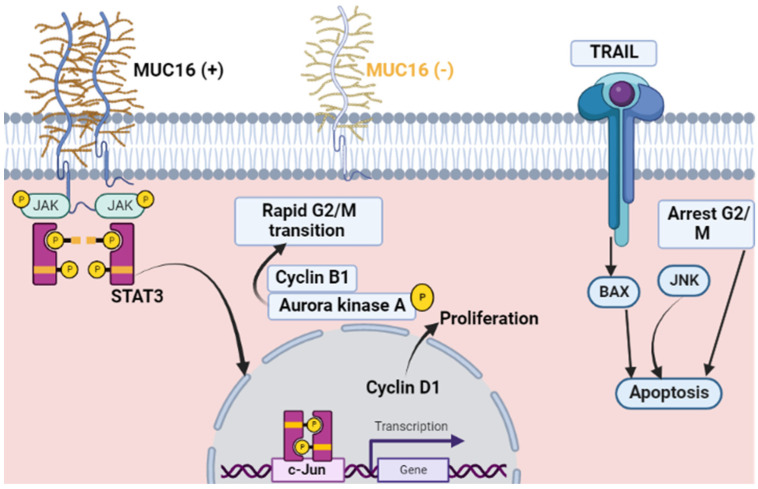
MUC16 expression in cancer: The MUC16 expression (+) stimulates tumor cell division via its binding to the non-receptor tyrosine kinase JAK2, which stimulates transcription factor STAT3 phosphorylation, which activates the expression of c-Jun for Cyclin D1. MUC16 influences the G2/M transition process via Cyclin B1 and phosphorylation of Aurora kinase A. Reduced expression of MUC16 (−) leads to malignant cells accumulation during the cell cycle’s G2/M phase with subsequent apoptosis of breast cancer cells via JNK signaling.

Thus, increased expression of mutated MUC16 promotes the proliferation and growth of lung cancer (Figure 1).

## 4. Muc16 (CA 125) and Metastasis

Numerous pulmonary cancers are linked to one or more distal spread, which accounts for 90% of all patient deaths [59]. Of note, lung cancer metastasis to the liver is observed in 5.8% of survivors.; though, the percentage with hepatic metastasis elevated meaningfully subsequent postmortem examination [44]. The liver is prone to be affected by lung cancer through blood circulation [44]. Tumor staging is usually attained by imaging examination that donates to TNM staging [60]. Although numerous promising medical imaging methods, like computed tomography and magnetic resonance imaging, significantly enhance the detection of metastasis, they still have some limitations [61]. Imaging methods, for instance, cannot detect occult micrometastases or some infiltrating hepatic lesions [61]. Additionally, some hepatic metastases that rapidly develop into fatal acute renal failure in SCLC individuals can only be observed in autopsy [62]. Many authors thought of independent prognostic indicators for the staging of lung cancer, as serological markers, tumor gene expression detection, and micrometastasis [63].

Furthermore, serological markers with high specificity and sensitivity for lung cancer metastasis to the liver have only been mentioned infrequently [63]. Different tumor markers have been widely used in clinical practice to diagnose lung cancer and predict patient prognosis. Nonetheless, these markers can be found in some benign tumors, resulting in a high false-negative/positive rate. Many researchers stated that the usage of combined tumor markers gives high sensitivity, but the best combination of such markers is still unknown. A combination of four tumor markers, CEA, CA 125, CYFRA 21-1, and squamous cell carcinoma, resulted in diagnostic sensitivity for lung cancer of 63.41% [64].

Therefore, the prediction of lung cancer metastasis to the liver, former to the presence of an imaged mass, would be a huge benefit for determining prognosis and developing personalized therapy [44]. Recently, CA125 was discovered to play a role in lymph nodes and peritoneal metastases [44]. Interestingly, CA125 serum levels can rise due to inflammation or metastasis [22]. Besides, CA125 levels were higher in patients with pancreatic and gastric cancer metastasis to the liver or peritoneum, and patients with higher baseline levels were more likely to relapse during the recovery period [65]. Although imaging was difficult in detecting small tumor metastases, CA125 is considered a significant serological marker [66]. Thus, CA125 levels were consistently linked to a poor prognosis and metastasis development, most likely because they promote tumor cell proliferation and suppress antitumor immune responses [40]. Even though levels of CA125 are related to tumor metastasis, organ specificity of this marker was argumentative among studies [40]. Remarkably, the concentration level of CA125 had been reported not to be linked with predicting the metastasis of the tumor to particular sites like the bone or liver [67]. It has been illustrated that CA125 expression levels varied obviously between the liver metastasis and non-liver metastasis groups [67], with satisfactory sensitivity and specificity. Unfortunately, patients with higher CA125 concentrations are more likely to have hematogenous tumor dissemination, and CA125 is an independent marker at the time of analysis, with a cutoff value of 13.65 U/mL [68]. The CA125 cutoff value is much higher (53 U/mL), supporting the hypothesis that liver metastases are mostly hematologic [69]. Numerous studies established that the CA125 level was meaningfully related to liver metastasis in other cancers [70,71]. Therefore, the CA125 serum level is regarded as an important tool for the prediction of liver metastasis of lung cancer [71]. It has been demonstrated that the elevation in CA125 levels in males was less clear than those in female patients, most likely because of female-specific organs, with the ovaries in particular having a great effect on serum levels of CA125 [72].

Moreover, the combination of NSE and CA125 has been shown to accurately predict liver metastasis, since NSE levels are highly diverse among individuals with all histological types of lung cancer with or without liver metastasis [44]. Individuals with elevated NSE levels in their serum are more likely to develop liver metastasis, implying that NSE levels could be a useful marker in the early stage [44]. Though NSE levels varied meaningfully among the two groups, NSE alone was insufficient in the prediction of liver metastasis due to its specificity being less than 90% [44]. Besides, the serum levels of CA125 are significantly greater in lung cancer individuals with liver metastasis than in those without, implying that CA125 has been linked to liver metastasis of lung cancer [73]. Both CA125 and NSE, in particular NSE, are more specific in liver metastasis [72,73]. Remarkably, the combined usage of the two markers gives greater specificity and sensitivity than either factor alone, indicating that this combination can achieve a more accurate prediction [44,73].

The final stage in the progression of lung cancer is liver metastasis, which is linked to a bad prognosis [74]. Even though several signs have been discovered as having predictive significance in lung cancer and liver metastasis, many patients still have liver metastases that are not detected by imaging [75]. Lung cancer patients (*n* = 1746) diagnosed between 2002 and 2016 were separated into two groups: those with liver metastases and those without. Calcium, CEA, CA125, and CA153 concentrations in the blood CA153, carbohydrate antigen-199 (CA199), neuron-specific antigen (NSA), total prostate-specific antigen (TPSA), and CYFRA21-1 are all examples of carbohydrate antigens. In both cases, NSE, CA199, neuron-specific antigen, TPSA, and CYFRA21-1 were studied [44]. In the two groups, no discernible difference was recorded in age or gender. CA125 and NSE were found to be substantially linked to liver metastases. NSE was more effective than CA125. It was less sensitive (*p* = 0.001), but more specific (*p* = 0.001). In individuals with non-alcoholic fatty liver disease, NSE concentrations were investigated further. NSE concentrations differed considerably between individuals with and without liver cancer in small-cell lung cancer patients (*p* = 0.023) metastasis [44].

Moreover, CA125 has been widely used to screen for tumors, particularly ovarian cancer. The serum CA125 level can be used as a better prognosis evaluation and it may dynamically monitor the disease progression [76]. The serum CA125 concentrations from 97 ovarian cancer cases revealed no association between age or CA125 concentration for the diagnosis and metastasis of ovarian cancer. Nevertheless, with the use of receiver operating characteristic (ROC) curves, the serum CA125 concentration cut-off value (82.9 U/mL) predicts metastasis. The area under the curve is 0.632. This cut-off value has the potential to be a helpful indicator for ovarian cancer metastasis [76]. In addition, CA125 is better than CA19-9 in the prediction of resectability, highlighting the possibility of a link between the serum concentration of CA125 and occult unresectable disease in patients with pancreatic cancer. Thus, serum CA125 may be useful as a pretreatment biomarker for tumor metastasis-associated burden in pancreatic tumors [77]. Therefore, serum CA125 levels are of great importance in the diagnosis and treatment of pancreatic tumors. CA125 concentration indicates the metastasis-associated burden of pancreatic cancer in individuals with advanced stages, in addition to the presence of occult metastasis in individuals with localized cancer. Using regular analysis of serum levels of CA125 in pre- and post-clinical assessments of pancreatic cancer treatment can upgrade treatment decisions and survival [77]. In addition, CA125 levels in the serum can be used to diagnose metastatic breast cancer, and combinations of different tumor markers have varying diagnostic values [78]. Remarkably, the CA125 level can predict the chance of surgical resection of the tumor as each week delay after the first CA125 elevation is associated with a 3% elevation in the opportunity of suboptimal resection at secondary cytoreductive surgery. Continuous monitoring of CA125 for early detection of recurrence may raise optimal SCS rates and influence overall survival [79].

Despite the important role of CA125 in the diagnosis and follow-up of lung cancer and other types of cancers, CA125 has some limitations in the diagnosis and prognosis of malignancies. One of the important limitations of CA125 is that up to 20% of ovarian cancers are devoid of antigen expression. Serum tumor markers found in ovarian cancers lacking CA125 expression may enhance the sensitivity for early detection [80]. CA125, like all tumor markers, is not specific to a particular tumor and may be increased in benign diseases. CA125 appears to be produced by mesothelial cells rather than cancer cells in ovarian cancer [81]. CA125 is a naturally occurring product of serosal epithelial cells and is found in the majority of serosal fluids, either malignant or benign. CA125 elevation can be caused by benign conditions, such as liver cirrhosis, peritoneal infection, abdominal surgery, or cardiac failure congestion. CA125 levels in the ascitic, pleural, or pericardial fluid of patients with cardiac failure are elevated, and serum levels are associated with the clinical staging of cardiac failure [81]. While CA125 levels can be used to diagnose cardiac failure, they can also be used to predict prognosis, especially when combined with natriuretic peptide measurements. The CA125 assay is not standardized and other assays, like new CA125II assays, frequently produce disparate results. Furthermore, as CA125 levels fall at menopause, and may rise in the elderly, reference limits appropriate for age and gender need to be refined for CA125 to fulfill any of its potential as a marker of cardiac failure in these age groups [80]. However, testing of CA125 in clinical practice may be associated with false-positive results. The CA125 sensitivity and specificity are not good and the Royal College of Pathologists or the Association of Clinical Biochemists did not produce any guidelines to help clinicians and laboratories in the appropriate use of CA125 [81]. The research included 799 individuals; 751 (94%) were women and 48 (6%) men; 221 (29%) women and 22 (46%) men had abnormal results. CA125 is primarily used to study various signs and symptoms, and few tests are used for ovarian cancer follow-up or screening. In patients with CA125 for probable malignancy/ovarian cancer, only 39 (20%) of the abnormal findings were due to ovarian cancer. Results with false-positive were mainly due to other malignancies (48 cases; 26%), benign ovarian disease (26 cases; 14%), and benign gynecologic diseases, especially leiomyoma (18 cases; 9%). CA125 specificity for ovarian cancer elevated at levels >1000 kU/L. These results confirm the high false-positive rate and low sensitivity and specificity associated with CA125. Significant inappropriate use of CA125 has resulted in unhelpful outcomes for clinicians, impacted costs, and increased patient anxiety and clinical uncertainty [81].

In comparison with other tumor biomarkers, in a study of 296 ovarian cancers, 65 (22%) showed mild or absent CA12 expression of CA125 by immunostaining. The expression of CA125 in tissue was compared to serum concentrations of CA125. Researchers discovered the presence of ten serum tumor markers in 65 epithelial ovarian carcinomas and cystadenomas, low-grade tumors, normal ovaries, and 16 other normal tissues with little or no CA125 expression by immunostaining. The absence of CA125 expression in surgical specimens of epithelial ovarian cancer was connected with low serum CA125 concentrations in pre-operative serum samples. Human kallikrein 10 (HK10), human kallikrein 6 (HK6), osteopontin (OPN), and claudin 3 were all expressed in ovarian cancers lacking CA125. The percentage of CA125-deficient ovarian cancers expressing DF3 (95%), vascular endothelial growth factor (VEGF) (81%), MUC1 (62%), mesothelin (MES) (34%), HE4 (32%), and CA19 is low-9 (29%). However, when reactivity with normal tissues was considered, MES and HE4 demonstrated the highest specificity. HK10, OPN, DF3, and MUC1 all showed differential expressions [80]. Each of the ten potential serum markers could be detected at the tissue level in 29–100% of ovarian cancers with low or absent CA125 expression. Many markers were found to be more abundant in cancers than in normal organs.

## 5. Mechanism of MUC16-Mediated Chemoresistance

MUC16 is implicated in ovarian cancer chemoresistance [82,83]; however, the mechanism of MUC16-mediated chemoresistance remains unknown. Cisplatin, a platinum analogue, is a DNA-damaging agent that is widely used in the treatment of lung cancer [84,85]. Similarly, gemcitabine is a nucleoside analogue commonly used to treat lung cancer patients [86]. MUC16 knockdown cells (both human and mouse tumors) were very sensitive to cisplatin and gemcitabine, while MUC16-Cter overexpressed cells were more resistant. These findings indicated that MUC16 may play a role in chemoresistance in cells of pulmonary cancer [2,87].

**Figure 2:**
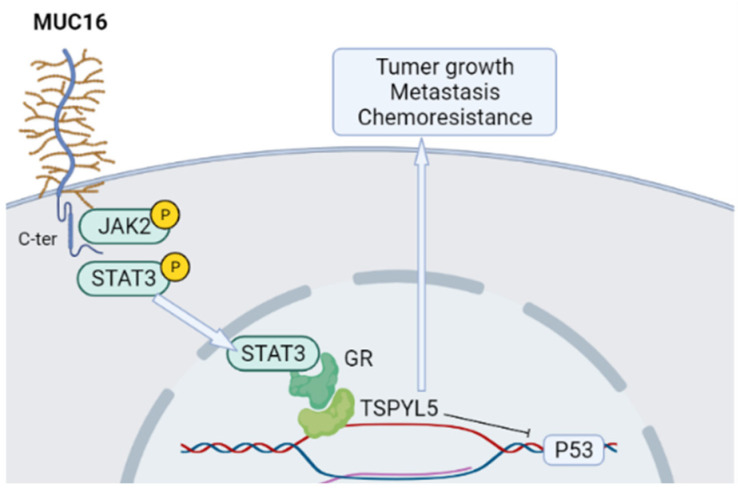
MUC16 chemoresistance signaling pathway in pulmonary tumor cells. MUC16 is essential for the phosphorylation of JAK2 and STAT3, with subsequent STAT3 translocation into the nucleus, in which it is responsible for recruitments of the glucocorticoids receptor (GR) that is responsible for the regulation of the TSPYL5 gene leading to tumor growth and metastasis. MUC16/TSPYL5 is responsible for p53 downregulation associated with chemoresistance. Created with BioRender.com.

The testis-specific Y-like protein 5 (TSPYL5) gene, located on chromosome 8q22, is frequently amplified in breast cancer and associated with poor prognosis [88]. TSPYL5 is implicated in the growth and metastasis of cancer cells. TSPYL5 binds with ubiquitin-specific protease 7 (USP7) and promotes the degradation of p53 to suppress tumor suppressor activity of p53 [88]. TSPYL5 has been implicated in cancer cell proliferation by activating Akt signaling and has been implicated in the radiation resistance of lung cancer cells [2]. Moreover, TSPYL5 overexpression suppresses the function of p53 and by regulating USP7, which causes the degradation of p53, its target genes [87]. In addition, TSPYL5 turned extensively downregulated in MUC16 knockdown cells. Likely, the expression of p53 and its target gene p21 was elevated in MUC16 knockdown cells. Furthermore, the expression of p53 was significantly downregulated in MUC16-Cter overexpressing cells compared with vector cells. In addition, marked p53 expression was detected in MUC16 knockdown cells (H292-shMUC16 seq1 and seq2) from transplanted xenograft tumor tissues with less tumor growth (Figure 2). Thus, MUC16 prevents p53 expression through TSPYL5 in cells of pulmonary cancer [2,46].

TSPYL5 knockdown in cells of pulmonary cancer led to an increment in p53 expression. These findings indicate that MUC16 inhibits p53 via TSPYL5 in cells of lung cancer. Furthermore, in cisplatin-resistant lung cancer cells, the expression of MUC16 was elevated, which strongly indicates the role of MUC16 in chemotherapeutic resistance in lung cancer. Overall, MUC16 regulates TSPYL5 with a subsequent decrease in the tumor suppressor activity of p53 [34,40], inducing lung cancer cell growth and chemoresistance. MUC16 knockdown cells (H292-shMUC16 seq1 and seq 2, H1975-shMUC16 seq1 and seq 2) were more sensitive to cisplatin and gemcitabine as shown by the MTT assay. In contrast, no significant alteration was detected in the untreated scramble (H292-SCR) and MUC16 knockdown (H292-shMUC16 seq1 and shMUC16 seq2) cells. Likely, MUC16-Cter overexpressing lung cancer cells (A549-F114HA) were more resistant to the cisplatin and gemcitabine cytotoxic effects [46,89]. Lakshmanan et al. [46] generated a mouse tumor cell line from genetically engineered mouse pulmonary cancer (Kras^G12D^; AdCre) tissues. The K1418 cell line has endogenous Muc16 and is stably knocked down by mouse Muc16-specific shRNA. MTT assays on these cell lines indicated that Muc16 knockdown (K1418-shMuc16) cells showed high sensitivity to cisplatin and gemcitabine. These findings suggest that MUC16 is responsible for the resistance of pulmonary carcinoma to cisplatin and gemcitabine (Figure 2).

Globally, lung cancer is considered the most common cancer with the highest incidence and mortalities, in particular pulmonary adenocarcinoma, SCLC, and lung squamous cell carcinoma. Cisplatin has the greatest importance in treating different subtypes of lung cancer, but this treatment is not beneficial for all individuals; thus, it is very important to determine the resistance or susceptibility of lung cancer patients to platinum-based therapy [90]. A panel of nine genes can precisely predict the sensitivity of patients to cisplatin, which can provide personalized treatment to lung cancer patients to improve their prognosis [90]. High levels of MUC16 in local residents could be one of the molecular characteristics of FLC. Surprisingly, individuals with higher MUC16 up-regulation appeared to have fewer white blood cells, particularly neutrophils; this indicates the role of MUC16 in immune regulation. The high levels of MUC16 in cell behavior experiments may be correlated with the proliferation, migration, invasion, and chemoresistance of lung cancer cells [53].

Thatcher et al. [86] formed cisplatin-resistant cell lines through exposure to diverse levels of cisplatin (100 nm–3.6 μM). The transcript of MUC16 was increased within cisplatin-resistant cell lines (*p* = 0.02) when matched with parental cells. These findings suggested that MUC16 plays a role in the chemoresistance to lung cancer. To determine the potential role of TSPYL5 in pulmonary cancer chemoresistance, they developed a stable knockdown of TSPYL5 in H292 lung cancer cells, and their results revealed that the expression of p53 was elevated in TSPYL5 knockdown cells when matched with scrambled cells.

## 6. Muc16 (CA 125) and Non-Malignant Proliferation

CA125 is released by embryonic cells [91]. It has recently been used in the prediction, diagnosis, and therapy of pulmonary cancer. The relation between CA125 and tumor size, stage, or histological type has not been studied [92]. In patients suffering from non-malignant lung cancer, it may be used as an independent prognostic indicator [93,94]. CA125 is also an effective method for diagnosing lung cancer patients with poor prognoses (PS). Benign pulmonary disease (BPD) is a type of lung disease for which PS tumor markers were not regularly evaluated on admission, and more PS patients were suspected to have higher tumor markers [95].

The level of CA125 increases in SCLC patients, but there is no significant difference between NSCLC, lung benign disease, and healthy people [96]. In addition, CA125 levels are linked to the TNM stage. CA125 levels were higher in stage IV than in stages I–III, and stage III was also higher than stage I [34]. In response to this, it was explained that a prior study showed that 84 patients (51 men and 33 females) living in air-polluted areas of China were measured previously. MUC16 mRNA levels were considerably higher in 48.8% (41/84) of NSCLC tissues as compared to matched adjacent noncancerous tissues. MUC16 mRNA expression, on the other hand, did not connect with gender, age, or histologic type [28,96]. Despite the small sample size, this study yielded positive results, demonstrating that gene mutations cause MUC16 upregulation [35,97].

An increase in CA125 levels also occurs in cases of benign cancers, including some gynecological cancers [98]. Several benign non-gynecological disorders, such as lung diseases including pulmonary tuberculosis and interstitial lung disease, have also been associated with significant increases in CA125 levels, and a relationship has been inferred between increased CA125 and the presence of excess fluid in the serous spaces [95,99].

As well, CA125 levels in the blood rise in both benign and malignant tumors, including ovarian, pancreatic, and colon cancers [99,100]. When the level of CA125 drops throughout treatment, it usually suggests that the cancer is responding. If the amount of CA125 does not change or rises following treatment, it could indicate that the cancer is not responding. After treatment, an increased CA125 result could indicate that cancer has returned [97,101]. One of the factors that affect the CA125 concentration is fluid effusion and retention. This is evident in patients with adenoma, where it was found that the CA125 concentration may reach 100 times its characteristic value [102]. Among the cases in which CA125 values are increased are lung diseases, including active tuberculosis, with values up to four times, the discriminatory value, which also increases to twice the discriminatory value in pneumonia, chronic obstructive pulmonary disease (COPD), lupus erythematosus, and interstitial lung disease [103,104,105].

Non-malignant lung tumor is diagnosed by the well-known method of early diagnosis of cancers, which is a high-resolution computerized tomography (HRCT). This imaging revealed the shape and site of the minute peripheral nodes [106]. However, chest radiography (CT) scans have limited usefulness in determining whether a lung lesion is benign or malignant [69]. Although a large volume of a tumor marker associated with lung cancer (e.g., CA125) usually indicates a diagnosis of benign lung cancer, sensitivity and specificity are insufficient in clinical practice for lung cancer screening and early diagnosis [21,107].

Circulating tumor cell (CTCs) detection can help distinguish between benign and malignant pulmonary nodules during screening for benign lung cancer [108]. In one study, a 68-year-old man had a 1.3-cm ground glass nodule identified during computerized tomography CT while being checked for a cough. Negative enrichment in situ hybridization (NEFISH) found 6 CTCs in this patient, but serum tumor markers, including CA125, were normal [109]. Pathologic diagnosis of the resulting sample after surgical resection showed early-stage lung cancer. A woman of 48 years was introduced to the hospital for right pulmonary space-occupying, and a 1.0-cm ground glass nodule was discovered in her right lung during a CT scan. Even though our method discovered 5 CTCs in this patient, all tumor markers in the serum were normal. This patient’s pathologic diagnosis after the operation showed lung cancer at early-stage (IA) [107]. These findings backed up the adoption of a 2 CTC threshold value for lung cancer diagnosis, indicating that this method outperforms traditional serum cancer indicators in terms of diagnostic efficacy [110]. CTCs and bronchoalveolar lavage fluid (BALF) had significantly better diagnostic performance than blood marker CA125 in distinguishing stage I lung cancer from benign lesions. The explanation for this could be that serum cancer markers are considered metabolic products that are regularly generated and secreted from cancerous cells and can be found in some benign tumors, increasing the percentage of false negatives and positives [107].

In many studies, the relationship between cancer antigens, such as CA125, and their effect on lung cancer patients, was clarified [109,111]. Panels of cancer serum antigens are established to increase the accuracy of diagnosis. A single panel of three sero antigens (CEA, CA125, cytokeratin fragment 21-1) and 1 autoantibody marker (New York esophageal squamous cell carcinoma 1) performed well in the high-risk group, with a sensitivity of 71% and specificity of 88% for lung cancer [112]. Forty clinical validations were carried out in a separate high-risk group (based on age and smoking history), yielding lower sensitivity (49%) but higher specificity (96%) [113]. Integration of clinical variables improved accuracy [36]. After the incorporation of variants including CA125, a series of 3144 asymptomatic individuals, of whom 1828 had lung cancer (52% stage IV), was performed [114]. The two-protein biomarker associated with clinical risk prediction for lung nodules was 97% sensitivity and 44% specificity. A total of 178 patients with suspicious lung nodules were evaluated for malignancy [115]. The performance of biomarkers was assessed for nodules with a pre-calculated risk of metastasis of 50% or less. For patients with benign nodules, this integrated classifier could have resulted in a 40% relative reduction in invasive testing (a 10% absolute risk reduction) with a 3% chance of delaying the management of malignant nodules [115,116].

## 7. CA125, Proteomics, and Genomics Findings

Proteomic analysis, a powerful tool for assessing protein expression, is widely used in tumor research. Quantitative protein expression profiling enables accurate and reproducible proper identification of differential expression levels of proteins across various biological samples [117]. Comparing protein expression profiles between normal tissues and cancers and between different cancers leads to the detection of tumor biomarkers, new therapeutic targets, and the illustration of molecular mechanisms of tumors [117]. The proteome is a set of proteins that an organism or system produces or modifies. Proteomics permits the identification of an increasing number of proteins. This depends on the time and various demands and stresses that cells and organisms are subjected to [117]. Several researchers studied the use of comparative proteomics in screening differentially expressed proteins in cell lines or lung cancer specimens [118,119]. It has been shown that isocitrate dehydrogenase 1, as a protein that promotes cancer growth, is a useful plasma marker for diagnosis and a histochemical marker predicting prognosis in NSCLC [117]. The combination of IDH1 expression and other prognostic factors like metastasis of lymph nodes, size of the tumor, differentiation, smoking habits, and gender may improve NSCLC prognosis prediction. Studies with larger sample sizes are required to develop a more reliable prognosis predictor, which will be estimated via the mathematical model of these prognosis-related variables [118]. For example, enzyme-linked immunosorbent assay (ELISA) analysis indicated that IDH1 plasma concentrations in the patients with NSCLC were significantly greater than those in patients with benign lung disease and healthy individuals [117]. Moore et al. [120] observed that proteomic biomarkers and CA125 demonstrated 84% sensitivity and 98% specificity for discriminating sera from patients who had stage I disease at the time of surgery, significantly overtaking the sensitivity of CA125 alone in women with epithelial ovarian cancer. Indeed, inter alpha-trypsin inhibitor heavy-chain 4 in combination with CA125 improved upon the sensitivity of CA125 alone for the detection of lung cancer at the early stage [121]. Moreover, the combined detection of autoantibodies against cytokeratin 19 fragment, CEA, and α-enolase improved the diagnostic sensitivity of NSCLC [122]. Therefore, autoantibodies against α-enolase could be a useful biomarker for NSCLC. Recently, Albanes et al. [123] observed that the detection of new risk biomarkers could improve early diagnosis of smoking-related lung cancer. As well, activation of proliferative signaling, tumor-promoted inflammation, and invasion and metastasis are most commonly detected in lung cancer pathogenesis [123]. Researchers detected 36 biomarkers of an impending smoking-related lung cancer diagnosis with a wide range of functions and relevance across cancer hallmarks after screening 1162 proteins [123]. In addition, the usefulness of circulating tumor DNA in guiding targeted therapy, predicting therapeutic response, and monitoring disease recurrence has been demonstrated. In advanced cancers, understanding, the origin of tissue, primary or metastatic lesions of ctDNA help to interpret its clinical importance more clearly. Moreover, ctDNA mutation patterns and their origins are still poorly studied [124]. Similarly, dynamic detection of ctDNA variant allele frequencies in plasma is of great value as a biomarker for assessing chemotherapy efficacy in patients with SCLC and advanced NSCLC, leading to earlier detection of lung cancer patients than radiography [125].

These findings suggest that proteomics and genomics findings are of great importance in the early detection and follow-up of pulmonary cancer.

## 8. Conclusions

For the past several decades, lung cancer is the most commonly diagnosed cancer in the world. Tumor of lung cancer is classified into two histological types: NSCLC and SCLC. NSCLC represents 80–85% of lung cancers, of which about 40% is adenocarcinoma, 25–30% is squamous cell carcinoma, and 10–15% is large cell carcinoma. In terms of the diagnosis of lung cancer, combination carcinoembryonic antigen (CEA) and cancer antigen 125 (CA125) detection had higher sensitivity, specificity, and diagnostic odds ratios than CEA detection alone. Most individuals with elevated serum CA125 levels had lung cancer that was either in stage 3 or stage 4. Serum CA125 levels were similarly elevated in lung cancer patients who also had pleural effusions or ascites. Furthermore, there is strong evidence that human lung cancer produces CA125 in vitro, which suggests that other clinical illnesses outside of ovarian cancer could also be responsible for the rise of CA125. MUC16 (CA125) is a natural killer cell inhibitor. As a screening test for the early diagnosis and prognosis of lung and ovarian cancer, CA125 has been widely employed as a biomarker in three different clinical settings. MUC16 mRNA levels in NSCLC tissues and adjacent non-malignant tissues are considerably higher in NSCLC tissues as compared to matched adjacent noncancerous tissues regardless of gender. Thus, CA125 could be a potential biomarker for the diagnosis and prognosis of lung cancer. As well, increased expression of mutated MUC16 promotes the proliferation and growth of lung cancer. In addition, the CA125 serum level is regarded as an important tool for the prediction of liver metastasis of lung cancer. Moreover, NSE and CA125 combination could help in the prediction of liver metastasis precisely, since NSE levels are highly diverse among individuals with all histological types of pulmonary cancer with or without hepatic metastasis. Further, CA125 could be a useful biomarker in other cancer types diagnoses like ovarian, breast, and pancreatic cancers. One of the important limitations of CA125 as a first step in such a screening technique is that up to 20% of ovarian tumors lack antigen expression. Each of the 10 possible serum markers was expressed in 29–100% of ovarian tumors with minimal or no CA125 expression. Thus, there is a controversy regarding CA125 in the diagnosis and prognosis of lung cancer and other cancer types. In this state, preclinical and clinical studies are warranted to elucidate the clinical benefit of CA125 in the diagnosis and prognosis of lung cancer.

## References

[1] Bade B.C., Dela Cruz C.S. (2020). Lung Cancer 2020: Epidemiology, Etiology, and Prevention. Clin. Chest Med..

[2] Kim E.J., Lee S.Y., Kim T.R., Im Choi S., Cho E.W., Kim K.C., Kim I.G. (2010). TSPYL5 is involved in cell growth and the resistance to radiation in A549 cells via the regulation of p21WAF1/Cip1 and PTEN/AKT pathway. Biochem. Biophys. Res. Commun..

[3] Schabath M.B., Cote M.L. (2019). Cancer progress and priorities: Lung cancer. Cancer Epidemiol. Biomark. Prev..

[4] Carter-Harris L., Slaven J.E., Monahan P.O., Shedd-Steele R., Hanna N., Rawl S.M. (2018). Understanding lung cancer screening behavior: Racial, gender, and geographic differences among Indiana long-term smokers. Prev. Med. Rep..

[5] Thandra K.C., Barsouk A., Saginala K., Aluru J.S., Barsouk A. (2021). Epidemiology of lung cancer. Contemp. Oncol./Współczesna Onkol..

[6] Lu T., Yang X., Huang Y., Zhao M., Li M., Ma K., Yin J., Zhan C., Wang Q. (2019). Trends in the incidence, treatment, and survival of patients with lung cancer in the last four decades. Cancer Manag. Res..

[7] Zhan X., Wang Z., Yang M., Luo Z., Wang Y., Li G. (2020). An electronic nose-based assistive diagnostic prototype for lung cancer detection with conformal prediction. Measurement.

[8] Takahashi S., Asada K., Takasawa K., Shimoyama R., Sakai A., Bolatkan A., Shinkai N., Kobayashi K., Komatsu M., Kaneko S. (2020). Predicting deep learning based multi-omics parallel integration survival subtypes in lung cancer using reverse phase protein array data. Biomolecules.

[9] De Sousa V.M.L., Carvalho L. (2018). Heterogeneity in lung cancer. Pathobiology.

[10] Hutchinson B.D., Shroff G.S., Truong M.T., Ko J.P. (2019). Spectrum of lung adenocarcinoma. Semin. Ultrasound CT MRI.

[11] Fidler-Benaoudia M.M., Torre L.A., Bray F., Ferlay J., Jemal A. (2020). Lung cancer incidence in young women vs. young men: A systematic analysis in 40 countries. Int. J. Cancer.

[12] Rusmaully J., Tvardik N., Martin D., Billmann R., Cénée S., Antoine M., Blons H., Laurent-Puig P., Trédaniel J., Wislez M. (2021). Risk of lung cancer among women in relation to lifetime history of tobacco smoking: A population-based case-control study in France (the WELCA study). BMC Cancer.

[13] Tanner N.T., Thomas N.A., Ward R., Rojewski A., Gebregziabher M., Toll B., Silvestri G.A. (2019). Association of cigarette type with lung cancer incidence and mortality: Secondary analysis of the National lung screening trial. JAMA Intern. Med..

[14] Schaal C.M., Bora-Singhal N., Kumar D.M., Chellappan S.P. (2018). Regulation of Sox2 and stemness by nicotine and electronic-cigarettes in non-small cell lung cancer. Mol. Cancer.

[15] Avino P., Scungio M., Stabile L., Cortellessa G., Buonanno G., Manigrasso M. (2018). Second-hand aerosol from tobacco and electronic cigarettes: Evaluation of the smoker emission rates and doses and lung cancer risk of passive smokers and vapers. Sci. Total Environ..

[16] Zahedi A., Phandthong R., Chaili A., Remark G., Talbot P. (2018). Epithelial-to-mesenchymal transition of A549 lung cancer cells exposed to electronic cigarettes. Lung Cancer.

[17] McAlinden K.D., Lu W., Eapen M.S., Sohal S.S. (2021). Electronic cigarettes: Modern instruments for toxic lung delivery and posing risk for the development of chronic disease. Int. J. Biochem. Cell Biol..

[18] Goldman J.W., Dvorkin M., Chen Y., Reinmuth N., Hotta K., Trukhin D., Statsenko G., Hochmair M.J., Özgüroğlu M., Ji J.H. (2021). Durvalumab, with or without tremelimumab, plus platinum–etoposide versus platinum–etoposide alone in first-line treatment of extensive-stage small-cell lung cancer (CASPIAN): Updated results from a randomised, controlled, open-label, phase 3 trial. Lancet Oncol..

[19] Howlader N., Forjaz G., Mooradian M.J., Meza R., Kong C.Y., Cronin K.A., Mariotto A.B., Lowy D.R., Feuer E.J. (2020). The effect of advances in lung-cancer treatment on population mortality. N. Engl. J. Med..

[20] Xie Y., Meng W.-Y., Li R.-Z., Wang Y.-W., Qian X., Chan C., Yu Z.-F., Fan X.-X., Pan H.-D., Xie C. (2021). Early lung cancer diagnostic biomarker discovery by machine learning methods. Transl. Oncol..

[21] Zang R., Li Y., Jin R., Wang X., Lei Y., Che Y., Lu Z., Mao S., Huang J., Liu C. (2019). Enhancement of diagnostic performance in lung cancers by combining CEA and CA125 with autoantibodies detection. Oncoimmunology.

[22] Clevers M.R., Kastelijn E.A., Peters B.J., Kelder H., Schramel F.M. (2021). Evaluation of serum biomarker CEA and Ca-125 as immunotherapy response predictors in metastatic non-small cell lung cancer. Anticancer Res..

[23] Homma S., Satoh H., Kagohashi K., Fujiwara M., Kamma H., Sekizawa K. (2004). Production of CA125 by human lung cancer cell lines. Clin. Exp. Med..

[24] Felder M., Kapur A., Gonzalez-Bosquet J., Horibata S., Heintz J., Albrecht R., Fass L., Kaur J., Hu K., Shojaei H. (2014). MUC16 (CA125): Tumor biomarker to cancer therapy, a work in progress. Mol. Cancer.

[25] Chen X.-K., Gu C.-L., Fan J.-Q., Zhang X.-M. (2020). P-STAT3 and IL-17 in tumor tissues enhances the prognostic value of CEA and CA125 in patients with lung adenocarcinoma. Biomed. Pharmacother..

[26] Zhang J., Han X., Gao C., Xing Y., Qi Z., Liu R., Wang Y., Zhang X., Yang Y.-G., Li X. (2018). 5-Hydroxymethylome in circulating cell-free DNA as a potential biomarker for non-small-cell lung cancer. Genom. Proteom. Bioinform..

[27] Zhang M., Zhang Y., Fu J., Zhang L. (2019). Serum CA125 levels are decreased in rectal cancer but increased in fibrosis-associated diseases and in most types of cancers. Prog. Mol. Biol. Transl. Sci..

[28] Bottoni P., Scatena R. (2015). The role of CA 125 as tumor marker: Biochemical and clinical aspects. Adv. Cancer Biomark..

[29] Masahashi T., Matsuzawa K., Ohsawa M., Narita O., Asai T., Ishihara M. (1988). Serum CA 125 levels in patients with endometriosis: Changes in CA 125 levels during menstruation. Obstet. Gynecol..

[30] Kafali H., Artunc H., Erdem M. (2007). Evaluation of factors that may be responsible for cyclic change of CA125 levels during menstrual cycle. Arch. Gynecol. Obstet..

[31] Dochez V., Caillon H., Vaucel E., Dimet J., Winer N., Ducarme G. (2019). Biomarkers and algorithms for diagnosis of ovarian cancer: CA125, HE4, RMI and ROMA, a review. J. Ovarian Res..

[32] Aithal A., Rauth S., Kshirsagar P., Shah A., Lakshmanan I., Junker W.M., Jain M., Ponnusamy M.P., Batra S.K. (2018). MUC16 as a novel target for cancer therapy. Expert Opin. Ther. Targets.

[33] Ma S., Shen L., Qian N., Chen K. (2012). The prognostic values of CA125, CA19. 9, NSE, AND SCC for stage I NSCLC are limited. Cancer Biomark..

[34] Kanwal M., Ding X.-J., Song X., Zhou G.-B., Cao Y. (2018). MUC16 overexpression induced by gene mutations promotes lung cancer cell growth and invasion. Oncotarget.

[35] Przepiorkowski J.A. (2021). The Association between Serum Cancer Antigen 125 (CA 125) and Risk of Lung Cancer in Females: Assessing the Possibilities for Early Detection. http://hdl.handle.net/10464/15097.

[36] Majewski S., Szewczyk K., Żal A., Białas A.J., Miłkowska-Dymanowska J., Piotrowski W.J. (2021). Serial Measurements of Circulating KL-6, SP-D, MMP-7, CA19-9, CA-125, CCL18, and Periostin in Patients with Idiopathic Pulmonary Fibrosis Receiving Antifibrotic Therapy: An Exploratory Study. J. Clin. Med..

[37] Albogami S.M., Al-kuraishy H.M., Al-Maiahy T.J., Al-Buhadily A.K., Al-Gareeb A.I., Alorabi M., Alotaibi S.S., De Waard M., Sabatier J.-M., Saad H.M. (2022). Hypoxia-Inducible Factor 1 and Preeclampsia: A New Perspective. Curr. Hypertens. Rep..

[38] Ram E., Lavee J., Raanani E., Patel J., Peled Y. (2021). Ca125 as an Early Marker for Graft Dysfunction in Antibody-Mediated Rejection: Guidance for Therapy. J. Heart Lung Transplant..

[39] Chen G., Wang Q., Wang K. (2022). MicroRNA-218-5p affects lung adenocarcinoma progression through targeting endoplasmic reticulum oxidoreductase 1 alpha. Bioengineered.

[40] Lei Y., Zang R., Lu Z., Zhang G., Huang J., Liu C., Wang Z., Mao S., Che Y., Wang X. (2020). ERO1L promotes IL6/sIL6R signaling and regulates MUC16 expression to promote CA125 secretion and the metastasis of lung cancer cells. Cell Death Dis..

[41] Lycke M., Ulfenborg B., Lauesgaard J.M., Kristjansdottir B., Sundfeldt K. (2021). Consideration should be given to smoking, endometriosis, renal function (eGFR) and age when interpreting CA125 and HE4 in ovarian tumor diagnostics. Clin. Chem. Lab. Med. (CCLM).

[42] Shafiq S., Zahan R., Yesmin S., Khan A., Mahmud M.S., Reza M.A., Albogami S.M., Alorabi M., De Waard M., Saad H.M. (2022). Phytochemical Analysis and Understanding the Antioxidant and Anticancer Properties of Methanol Extract from Litsea glutinosa: In Vitro and In Vivo Studies. Molecules.

[43] Wang Z., Huang J., Wang M., Bi W., Fan T. (2022). Analysis on the Effects of CT-and Ultrasound-Guided Percutaneous Transthoracic Needle Biopsy Combined with Serum CA125 and CEA on the Diagnosis of Lung Cancer. J. Healthc. Eng..

[44] Wang C.-F., Peng S.-J., Liu R.-Q., Yu Y.-J., Ge Q.-M., Liang R.-B., Li Q.-Y., Li B., Shao Y. (2020). The combination of CA125 and NSE is useful for predicting liver metastasis of lung cancer. Dis. Markers.

[45] Cong L., Feng W., Yao Z., Zhou X., Xiao W. (2020). Deep learning model as a new trend in computer-aided diagnosis of tumor pathology for lung cancer. J. Cancer.

[46] Lakshmanan I., Salfity S., Seshacharyulu P., Rachagani S., Thomas A., Das S., Majhi P.D., Nimmakayala R.K., Vengoji R., Lele S.M. (2017). MUC16 Regulates TSPYL5 for Lung Cancer Cell Growth and Chemoresistance by Suppressing p53MUC16, TSPYL5, and Lung Cancer. Clin. Cancer Res..

[47] Kashf D.W.A., Okasha A.N., Sahyoun N.A., El-Rabi R.E., Abu-Naser S.S. (2018). Predicting DNA lung cancer using artificial neural network. Int. J. Acad. Dev..

[48] Giamougiannis P., Martin-Hirsch P.L., Martin F.L. (2021). The evolving role of MUC16 (CA125) in the transformation of ovarian cells and the progression of neoplasia. Carcinogenesis.

[49] Khanmohammadi A., Aghaie A., Vahedi E., Qazvini A., Ghanei M., Afkhami A., Hajian A., Bagheri H. (2020). Electrochemical biosensors for the detection of lung cancer biomarkers: A review. Talanta.

[50] White B., Patterson M., Karnwal S., Brooks C.L. (2022). Crystal structure of a human MUC16 SEA domain reveals insight into the nature of the CA125 tumor marker. Proteins Struct. Funct. Bioinform..

[51] Charkhchi P., Cybulski C., Gronwald J., Wong F.O., Narod S.A., Akbari M.R. (2020). CA125 and ovarian cancer: A comprehensive review. Cancers.

[52] Funston G., Hamilton W., Abel G., Crosbie E.J., Rous B., Walter F.M. (2020). The diagnostic performance of CA125 for the detection of ovarian and non-ovarian cancer in primary care: A population-based cohort study. PLoS Med..

[53] Chen Y., Huang Y., Kanwal M., Li G., Yang J., Niu H., Li Z., Ding X. (2019). MUC16 in non-small cell lung cancer patients affected by familial lung cancer and indoor air pollution: Clinical characteristics and cell behaviors. Transl. Lung Cancer Res..

[54] Zhang L., Han X., Shi Y. (2020). Association of MUC16 Mutation With Response to Immune Checkpoint Inhibitors in Solid Tumors. JAMA Netw. Open.

[55] Barta J.A., Powell C.A., Wisnivesky J.P. (2019). Global Epidemiology of Lung Cancer. Ann. Glob. Health.

[56] Van Meerbeeck J.P., Fennell D.A., De Ruysscher D.K. (2011). Small-cell lung cancer. Lancet.

[57] Gao Y., Goldstein A.M., Consonni D., Pesatori A.C., Wacholder S., Tucker M.A., Caporaso N.E., Goldin L., Landi M.T. (2009). Family history of cancer and nonmalignant lung diseases as risk factors for lung cancer. Int. J. Cancer.

[58] Leonetti A., Wever B., Mazzaschi G., Assaraf Y.G., Rolfo C., Quaini F., Tiseo M., Giovannetti E. (2019). Molecular basis and rationale for combining immune checkpoint inhibitors with chemotherapy in non-small cell lung cancer. Drug Resist. Updat. Rev. Comment. Antimicrob. Anticancer Chemother..

[59] Chang J.Y., Senan S., Paul M.A., Mehran R.J., Louie A.V., Balter P., Groen H.J., McRae S.E., Widder J., Feng L. (2015). Stereotactic ablative radiotherapy versus lobectomy for operable stage I non-small-cell lung cancer: A pooled analysis of two randomised trials. Lancet Oncol..

[60] Lakshmanaprabu S., Mohanty S.N., Shankar K., Arunkumar N., Ramirez G. (2019). Optimal deep learning model for classification of lung cancer on CT images. Future Gener. Comput. Syst..

[61] Nishio M., Sugiyama O., Yakami M., Ueno S., Kubo T., Kuroda T., Togashi K. (2018). Computer-aided diagnosis of lung nodule classification between benign nodule, primary lung cancer, and metastatic lung cancer at different image size using deep convolutional neural network with transfer learning. PLoS ONE.

[62] Dammas S., Patz E.F., Goodman P.C. (2001). Identification of small lung nodules at autopsy: Implications for lung cancer screening and overdiagnosis bias. Lung Cancer.

[63] Shin J., Song S.-Y., Ahn H.-S., An B.C., Choi Y.-D., Yang E.G., Na K.-J., Lee S.-T., Park J.-I., Kim S.-Y. (2017). Integrative analysis for the discovery of lung cancer serological markers and validation by MRM-MS. PLoS ONE.

[64] Chen Z.-Q., Huang L.-S., Zhu B. (2018). Assessment of seven clinical tumor markers in diagnosis of non-small-cell lung cancer. Dis. Mrk..

[65] Hwang G.I., Yoo C.H., Sohn B.H., Shin J.H., Park Y.L., Dai Kim H., Kim Y.S., Han W.K., Pae W.K. (2004). Predictive value of preoperative serum CEA, CA19-9 and CA125 levels for peritoneal metastasis in patients with gastric carcinoma. Cancer Res. Treat. Off. J. Korean Cancer Assoc..

[66] Huang H., Yang Y., Zhu Y., Chen H., Yang Y., Zhang L., Li W. (2022). Blood protein biomarkers in lung cancer. Cancer Lett..

[67] Wang J., Chu Y., Li J., Zeng F., Wu M., Wang T., Sun L., Chen Q., Wang P., Zhang X. (2020). Development of a prediction model with serum tumor markers to assess tumor metastasis in lung cancer. Cancer Med..

[68] Ge Q.-M., Zou Y.-T., Shi W.-Q., Zhang Y.-Q., Li B., Min Y.-L., Yuan Q., Shao Y. (2020). Ocular Metastasis in Elderly Lung Cancer Patients: Potential Risk Factors of CA-125, CA-153 and TPSA. Cancer Manag. Res..

[69] Yang Q., Zhang P., Wu R., Lu K., Zhou H. (2018). Identifying the best marker combination in CEA, CA125, CY211, NSE, and SCC for lung cancer screening by combining ROC curve and logistic regression analyses: Is it feasible?. Dis. Markers.

[70] Zhang Z., Yuan F., Chen R., Li Y., Ma J., Yan X., Wang L., Zhang F., Tao H., Guo D. (2020). Dynamics of serum tumor markers can serve as a prognostic biomarker for Chinese advanced non-small cell lung cancer patients treated with immune checkpoint inhibitors. Front. Immunol..

[71] Huang L.L., Hu X.S., Wang Y., Li J.L., Wang H.Y., Liu P., Xu J.P., He X.H., Hao X.Z., Jiang P.D. (2021). Survival and pretreatment prognostic factors for extensive-stage small cell lung cancer: A comprehensive analysis of 358 patients. Thorac. Cancer.

[72] Chanvorachote P., Luanpitpong S., Chunhacha P., Promden W., Sriuranpong V. (2012). Expression of CA125 and cisplatin susceptibility of pleural effusion-derived human lung cancer cells from a Thai patient. Oncol. Lett..

[73] Li Z., Zhao J. (2021). Clinical efficacy and safety of crizotinib and alectinib in ALK-positive non-small cell lung cancer treatment and predictive value of CEA and CA125 for treatment efficacy. Am. J. Transl. Res..

[74] Shan Y., Ma J., Pan Y., Hu J., Liu B., Jia L. (2018). LncRNA SNHG7 sponges miR-216b to promote proliferation and liver metastasis of colorectal cancer through upregulating GALNT1. Cell Death Dis..

[75] Yu J., Green M.D., Li S., Sun Y., Journey S.N., Choi J.E., Rizvi S.M., Qin A., Waninger J.J., Lang X. (2021). Liver metastasis restrains immunotherapy efficacy via macrophage-mediated T cell elimination. Nat. Med..

[76] Yuan Q., Song J., Yang W., Wang H., Huo Q., Yang J., Yu X., Liu Y., Xu C., Bao H. (2017). The effect of CA125 on metastasis of ovarian cancer: Old marker new function. Oncotarget.

[77] Luo G., Xiao Z., Long J., Liu Z., Liu L., Liu C., Xu J., Ni Q., Yu X. (2013). CA125 is superior to CA19-9 in predicting the resectability of pancreatic cancer. J. Gastrointest. Surg..

[78] Wang W., Xu X., Tian B., Wang Y., Du L., Sun T., Shi Y., Zhao X., Jing J. (2017). The diagnostic value of serum tumor markers CEA, CA19-9, CA125, CA15-3, and TPS in metastatic breast cancer. Clin. Chim. Acta.

[79] Fleming N.D., Cass I., Walsh C.S., Karlan B.Y., Li A.J. (2011). CA125 surveillance increases optimal resectability at secondary cytoreductive surgery for recurrent epithelial ovarian cancer. Gynecol. Oncol..

[80] Rosen D.G., Wang L., Atkinson J.N., Yu Y., Lu K.H., Diamandis E.P., Hellstrom I., Mok S.C., Liu J., Bast R.C. (2005). Potential markers that complement expression of CA125 in epithelial ovarian cancer. Gynecol. Oncol..

[81] Moss E., Hollingworth J., Reynolds T. (2005). The role of CA125 in clinical practice. J. Clin. Pathol..

[82] Nowak M., Klink M. (2020). The Role of Tumor-Associated Macrophages in the Progression and Chemoresistance of Ovarian Cancer. Cells.

[83] Gardner G.J., Baser R.E., Brady M.F., Bristow R.E., Markman M., Spriggs D., Thaler H.T. (2012). CA125 regression in ovarian cancer patients treated with intravenous versus intraperitoneal platinum-based chemotherapy: A gynecologic oncology group study. Gynecol. Oncol..

[84] Gatzemeier U., von Pawel J., Vynnychenko I., Zatloukal P., de Marinis F., Eberhardt W.E., Paz-Ares L., Schumacher K.M., Goddemeier T., O’Byrne K.J. (2011). First-cycle rash and survival in patients with advanced non-small-cell lung cancer receiving cetuximab in combination with first-line chemotherapy: A subgroup analysis of data from the FLEX phase 3 study. Lancet Oncol..

[85] Pless M., Stupp R., Ris H.B., Stahel R.A., Weder W., Thierstein S., Gerard M.A., Xyrafas A., Früh M., Cathomas R. (2015). Induction chemoradiation in stage IIIA/N2 non-small-cell lung cancer: A phase 3 randomised trial. Lancet.

[86] Thatcher N., Hirsch F.R., Luft A.V., Szczesna A., Ciuleanu T.E., Dediu M., Ramlau R., Galiulin R.K., Bálint B., Losonczy G. (2015). Necitumumab plus gemcitabine and cisplatin versus gemcitabine and cisplatin alone as first-line therapy in patients with stage IV squamous non-small-cell lung cancer (SQUIRE): An open-label, randomised, controlled phase 3 trial. Lancet Oncol..

[87] Epping M.T., Meijer L.A., Krijgsman O., Bos J.L., Pandolfi P.P., Bernards R. (2011). TSPYL5 suppresses p53 levels and function by physical interaction with USP7. Nat. Cell Biol..

[88] Van ’t Veer L.J., Dai H., van de Vijver M.J., He Y.D., Hart A.A., Mao M., Peterse H.L., van der Kooy K., Marton M.J., Witteveen A.T. (2002). Gene expression profiling predicts clinical outcome of breast cancer. Nature.

[89] Pal S.K., Frankel P.H., Mortazavi A., Milowsky M., Vaishampayan U., Parikh M., Lyou Y., Weng P., Parikh R., Teply B. (2021). Effect of Cisplatin and Gemcitabine With or Without Berzosertib in Patients With Advanced Urothelial Carcinoma: A Phase 2 Randomized Clinical Trial. JAMA Oncol..

[90] Gao Y., Lyu Q., Luo P., Li M., Zhou R., Zhang J., Lyu Q. (2021). Applications of Machine Learning to Predict Cisplatin Resistance in Lung Cancer. Int. J. Gen. Med..

[91] Chen Q., Lu Q., Fei X., Li C., Li B. (2021). Elevated tumor markers in a benign lung disease. J. Cardiothorac. Surg..

[92] Li J., Liu L., Feng Z., Wang X., Huang Y., Dai H., Zhang L., Song F., Wang D., Zhang P. (2020). Tumor markers CA15-3, CA125, CEA and breast cancer survival by molecular subtype: A cohort study. Breast Cancer.

[93] Yu D., Du K., Liu T., Chen G. (2013). Prognostic value of tumor markers, NSE, CA125 and SCC, in operable NSCLC Patients. Int. J. Mol. Sci..

[94] Wang L., Wang D., Zheng G., Yang Y., Du L., Dong Z., Zhang X., Wang C. (2016). Clinical evaluation and therapeutic monitoring value of serum tumor markers in lung cancer. Int. J. Biol. Markers.

[95] Tahmasebi F., Nath R., Sokolovsky N., Scaffidi J., Boley J., Mehra G., Sayanseh A. (2018). Incidental finding of raised CA125: A cause for concern. Crit Care Obs. Gyne.

[96] Yuan M., Huang L.-L., Chen J.-H., Wu J., Xu Q. (2019). The emerging treatment landscape of targeted therapy in non-small-cell lung cancer. Signal. Transduct. Target. Ther..

[97] Sears C.R., Mazzone P.J. (2020). Biomarkers in lung cancer. Clin. Chest Med..

[98] Wang Q., Peng H., Qi X., Wu M., Zhao X. (2020). Targeted therapies in gynecological cancers: A comprehensive review of clinical evidence. Signal. Transduct. Target. Ther..

[99] Fraser C.C., Jia B., Hu G., Al Johani L.I., Fritz-Klaus R., Ham J.D., Fichorova R.N., Elias K.M., Cramer D.W., Patankar M.S. (2022). Ovarian Cancer Ascites Inhibits Transcriptional Activation of NK Cells Partly through CA125. J. Immunol..

[100] Radhakrishnan A., Malukar N., Jain S. (2020). Serum CA-125 and Serum CEA Ratio to Distinguish between Ovarian Malignancies and Non-ovarian Malignancies. Indian J. Med. Biochem..

[101] Gunn A.H., Tashie C., Wolf S., Troy J.D., Zafar Y. (2022). Tumor marker response to SARS-CoV-2 infection among patients with cancer. Cancer Med..

[102] Singha B., Harper S.L., Goldman A.R., Bitler B.G., Aird K.M., Borowsky M.E., Cadungog M.G., Liu Q., Zhang R., Jean S. (2018). CLIC1 and CLIC4 complement CA125 as a diagnostic biomarker panel for all subtypes of epithelial ovarian cancer. Sci. Rep..

[103] Tansir G., Kumar P., Pius A., Sunny S., Soneja M. (2019). Pseudo-pseudo Meigs’ syndrome: A rare presentation of systemic lupus erythematosus. Reumatismo.

[104] Bao Y., Zhang W., Shi D., Bai W., He D., Wang D. (2021). Correlation between serum tumor marker levels and connective tissue disease-related interstitial lung disease. Int. J. Gen. Med..

[105] Trape J., Filella X., Alsina-Donadeu M., Juan-Pereira L., Bosch-Ferrer A., Rigo-Bonnin R., Oncology Section of the Catalan Association of Clinical Laboratory Science (2011). Increased plasma concentrations of tumour markers in the absence of neoplasia. Clin. Chem. Lab. Med..

[106] Marsaa K., Gundestrup S., Jensen J.-U., Lange P., Løkke A., Roberts N.B., Shaker S.B., Sørensen A.R., Titlestad I.L., Thomsen L.H. (2018). Danish respiratory society position paper: Palliative care in patients with chronic progressive non-malignant lung diseases. Eur. Clin. Respir. J..

[107] Zhong C.-H., Tong D., Zhou Z.-Q., Su Z.-Q., Luo Y.-L., Xing J., Bai Y.-L., Guo S.-J., Li S.-Y. (2018). Performance evaluation of detecting circulating tumor cells and tumor cells in bronchoalveolar lavage fluid in diagnosis of peripheral lung cancer. J. Thorac. Dis..

[108] Zhang X., Li H., Yu X., Li S., Lei Z., Li C., Zhang Q., Han Q., Li Y., Zhang K. (2018). Analysis of circulating tumor cells in ovarian cancer and their clinical value as a biomarker. Cell. Physiol. Biochem..

[109] Guo Y.-X., Neoh K.H., Chang X.-H., Sun Y., Cheng H.-Y., Ye X., Ma R.-Q., Han R.P., Cui H. (2018). Diagnostic value of HE4+ circulating tumor cells in patients with suspicious ovarian cancer. Oncotarget.

[110] Li Y., Tian X., Gao L., Jiang X., Fu R., Zhang T., Ren T., Hu P., Wu Y., Zhao P. (2019). Clinical significance of circulating tumor cells and tumor markers in the diagnosis of lung cancer. Cancer Med..

[111] Wang D., Yang Y., Jin L., Wang J., Zhao X., Wu G., Zhang J., Kou T., Yao H., Zhang Z. (2019). Prognostic models based on postoperative circulating tumor cells can predict poor tumor recurrence-free survival in patients with stage II-III colorectal cancer. J. Cancer.

[112] Doseeva V., Colpitts T., Gao G., Woodcock J., Knezevic V. (2015). Performance of a multiplexed dual analyte immunoassay for the early detection of non-small cell lung cancer. J. Transl. Med..

[113] Mazzone P.J., Wang X.F., Han X., Choi H., Seeley M., Scherer R., Doseeva V. (2018). Evaluation of a Serum Lung Cancer Biomarker Panel. Biomark Insights.

[114] Molina R., Marrades R.M., Augé J.M., Escudero J.M., Viñolas N., Reguart N., Ramirez J., Filella X., Molins L., Agustí A. (2016). Assessment of a combined panel of six serum tumor markers for lung cancer. Am. J. Respir. Crit. Care Med..

[115] Seijo L.M., Peled N., Ajona D., Boeri M., Field J.K., Sozzi G., Pio R., Zulueta J.J., Spira A., Massion P.P. (2019). Biomarkers in lung cancer screening: Achievements, promises, and challenges. J. Thorac. Oncol..

[116] Silvestri G.A., Tanner N.T., Kearney P., Vachani A., Massion P.P., Porter A., Springmeyer S.C., Fang K.C., Midthun D., Mazzone P.J. (2018). Assessment of plasma proteomics biomarker’s ability to distinguish benign from malignant lung nodules: Results of the PANOPTIC (Pulmonary Nodule Plasma Proteomic Classifier) trial. Chest.

[117] Tan F., Jiang Y., Sun N., Chen Z., Lv Y., Shao K., Li N., Qiu B., Gao Y., Li B. (2012). Identification of isocitrate dehydrogenase 1 as a potential diagnostic and prognostic biomarker for non-small cell lung cancer by proteomic analysis. Mol. Cell. Proteom..

[118] Poschmann G., Sitek B., Sipos B., Ulrich A., Wiese S., Stephan C., Warscheid B., Klöppel G., Vander Borght A., Ramaekers F.C. (2009). Identification of proteomic differences between squamous cell carcinoma of the lung and bronchial epithelium. Mol. Cell. Proteom..

[119] Seike M., Kondo T., Fujii K., Okano T., Yamada T., Matsuno Y., Gemma A., Kudoh S., Hirohashi S. (2005). Proteomic signatures for histological types of lung cancer. Proteomics.

[120] Moore L.E., Pfeiffer R.M., Zhang Z., Lu K.H., Fung E.T., Bast R.C. (2012). Proteomic biomarkers in combination with CA 125 for detection of epithelial ovarian cancer using prediagnostic serum samples from the Prostate, Lung, Colorectal, and Ovarian (PLCO) Cancer Screening Trial. Cancer.

[121] Zhang Z., Bast R.C., Yu Y., Li J., Sokoll L.J., Rai A.J., Rosenzweig J.M., Cameron B., Wang Y.Y., Meng X.Y. (2004). Three biomarkers identified from serum proteomic analysis for the detection of early stage ovarian cancer. Cancer Res..

[122] He P., Naka T., Serada S., Fujimoto M., Tanaka T., Hashimoto S., Shima Y., Yamadori T., Suzuki H., Hirashima T. (2007). Proteomics-based identification of alpha-enolase as a tumor antigen in non-small lung cancer. Cancer Sci..

[123] Albanes D., Alcala K., Alcala N., Amos C., Arslan A., Bassett J., Brennan P., Cai Q., Chen C., Feng X. (2022). The Blood Proteome of Imminent Lung Cancer Diagnosis. medRxiv.

[124] Fu R., Tang W.-F., Yang L.-L., Wu M., Bao H., Shao Y., Zhang C., Hong H.-Z., Wu Y.-L., Zhong W.-Z. (2022). EP16. 02-024 Plasma ctDNA Organ-Specific Genomic Patterns and Origination Analysis in Advanced Non-Small Cell Lung Cancer. J. Thorac. Oncol..

[125] Zhang M., Huang C., Zhou H., Liu D., Chen R., Li X., Cheng Y., Gao B., Chen J. (2022). Circulating tumor DNA predicts the outcome of chemotherapy in patients with lung cancer. Thorac. Cancer.

